# Eurasian house mouse (*Mus musculus* L.) differentiation at microsatellite loci identifies the Iranian plateau as a phylogeographic hotspot

**DOI:** 10.1186/s12862-015-0306-4

**Published:** 2015-02-25

**Authors:** Emilie A Hardouin, Annie Orth, Meike Teschke, Jamshid Darvish, Diethard Tautz, François Bonhomme

**Affiliations:** Max Planck Institute of Evolutionary Biology, Department of Evolutionary Genetics, August-Thienemann-Str. 2, 24306 Plön, Germany; Department of Life and Environmental Sciences, Faculty of Science and Technology, Bournemouth University, Christchurch House, Talbot Campus, Poole, Dorset BH12 5BB UK; Institut des Sciences de l’Evolution, CNRS, IRD, EPHE, Université de Montpellier, Pl. E. Bataillon, 34095 Montpellier, France; Rodentology Research group, Ferdowsi University of Mashhad, Mashhad, Iran

**Keywords:** House mouse, Microsatellites, Phylogeographic hotspot, Iranian plateau

## Abstract

**Background:**

The phylogeography of the house mouse (*Mus musculus* L.), an emblematic species for genetic and biomedical studies, is only partly understood, essentially because of a sampling bias towards its most peripheral populations in Europe, Asia and the Americas. Moreover, the present-day phylogeographic hypotheses stem mostly from the study of mitochondrial lineages. In this article, we complement the mtDNA studies with a comprehensive survey of nuclear markers (19 microsatellite loci) typed in 963 individuals from 47 population samples, with an emphasis on the putative Middle-Eastern centre of dispersal of the species.

**Results:**

Based on correspondence analysis, distance and allele-sharing trees, we find a good coherence between geographical origin and genetic make-up of the populations. We thus confirm the clear distinction of the three best described peripheral subspecies, *M. m. musculus*, *M. m. domesticus* and *M. m. castaneus*. A large diversity was found in the Iranian populations, which have had an unclear taxonomic status to date. In addition to samples with clear affiliation to *M. m. musculus* and *M. m. domesticus*, we find two genetic groups in Central and South East Iran, which are as distinct from each other as they are from the south-east Asian *M. m. castaneus*. These groups were previously also found to harbor distinct mitochondrial haplotypes.

**Conclusion:**

We propose that the Iranian plateau is home to two more taxonomic units displaying complex primary and secondary relationships with their long recognized neighbours. This central region emerges as the area with the highest known diversity of mouse lineages within a restricted geographical area, designating it as the focal place to study the mechanisms of speciation and diversification of this species.

**Electronic supplementary material:**

The online version of this article (doi:10.1186/s12862-015-0306-4) contains supplementary material, which is available to authorized users.

## Background

The house mouse *(Mus musculus* L.) has long been viewed has an excellent model for the study of evolution and its genome has been one of the first to be nearly completely sequenced [1,2]. Moreover, its dispersal capacity through commensalism has ranked it as one of the “100 world worst most invasive alien species” (ISSG), therefore offering various possibilities to study adaptation to various environments [3]. At the same time, this species is one of the most studied vertebrates due to its use as a prominent laboratory model, but its phylogeography and population genetics is so far only partly understood [4]. The current knowledge has slowly accumulated over the last 30 years in a non-optimal fashion, since its most peripheral populations in Europe, Asia and the Americas have been studied before insights were gained for those from the Middle-Eastern centre of its distribution [5].

It is now widely recognised that *Mus musculus* L. constitutes a complex assembly of more or less well separated populations and subspecies. The term “subspecies” in itself is taken here in its broad sense of “genetically recognisable entities” but this does not imply on our part any deeper statement about the actual level of isolation among these entities. The last 45 years of literature on systematics of the house mouse revealed that nomenclatorial issues have been quite controversial, with the use of many terms ranging from biochemical groups, subspecies, semi-species to full species to designate the same entities. Here, we follow the generally held view that the more widely distributed populations are grouped into three different subspecies: *Mus musculus musculus* in Eastern Europe, Central and North East Asia, *Mus musculus domesticus* in Northern Africa and Western Europe, and *Mus musculus castaneus* in South East Asia. These last two subspecies have further expanded in modern times to the Americas, Australia and Oceania [6-9]. In addition, *Mus musculus molossinus,* a hybrid between *M. m. musculus* and *M. m. castaneus* found in Japan [10] is often considered as a subspecies on its own. Closer to the centre of the distribution, *Mus musculus gentilulus* has been identified in the eastern part of the Arabic peninsula on the basis of its mitochondrial DNA lineage [11] while from the same type of data [12,13] it has been shown that certain populations considered as *M. m. castaneus* in Iran, Pakistan and Afghanistan should probably be considered as belonging to further sub-specific groups. Moreover, another completely independent lineage has recently been identified on this basis in Nepal [13]. Hence, the taxonomic situation close to the Middle-Eastern centre is far from being fully clarified. Since taxonomy reflects history, this clarification is a prerequisite if we want to further study the evolutionary mechanisms accounting for the species’ differentiation.

**Figure 1:**
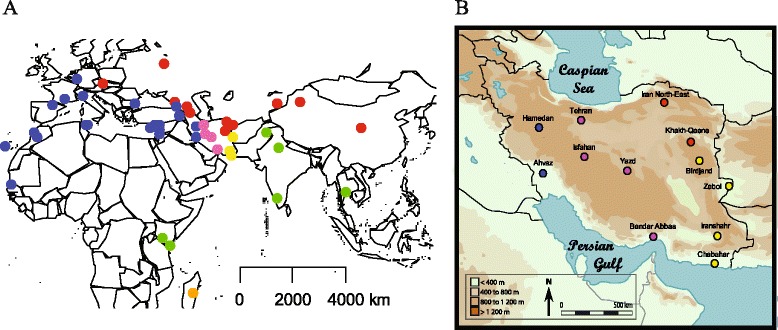
**Location of the house mouse samples used in this study.** Blue dots represent *M. m. domesticus* population, red dots *M. m. musculus*, green dots *M. m. castaneus*, pink dots central Iran, yellow dots South East Iran and orange dots Madagascar. **A)** World view, **B)** Map of Iran (adapted from [14]).

The present study aims at filling this gap through the analysis of genetic variation at nuclear loci and is the first attempt to directly compare a set of population samples covering most of the Eurasian distribution of the species. We report on the variability at 19 microsatellite loci typed in 963 individuals originating from 47 populations in Europe, Asia, Africa and the Middle-East (Figure 1).

Our main findings support the global picture of a species having initially differentiated in several genetic entities in the Middle-East forming the extant subspecies. Several of these subsequently expanded outwards because of their propensity to engage in commensalism and finally colonise the entire world. Of particular interest is the situation of the populations inhabiting the Iranian plateau where, despite an important level of secondary admixture in this region, four main genetic groups could be identified, in partial congruence with the mitochondrial analysis.

## Results

### Genetic diversity

The genetic diversity was calculated for all samples (Table 1). The most diverse sample was the one from Ahvaz in Iran with a H_exp_ of 0.89 and an average number of alleles across loci of 19.3. The lowest H_exp_ is seen for Moscow (0.49) with only 3.1 alleles/loci, but this can be ascribed to the small sample size (N = 5). Among the three islands that are present in this study, La Palma (Canaries) and Cyprus displayed a H_exp_ comparable to continental populations (0.74 and 0.77 respectively), while the Madagascar value was slightly lower (0.67). Mean and Median of H_exp_ are 0.74 and 0.75 respectively. The global differentiation as measured by inter-sample F_ST_ was = 0.17, albeit non-uniformly distributed among the 19 loci, with some loci contributing more than others. There are two outlier loci displaying global F_ST_ values of 0.52 (D1EnsmusG22992) and 0.41 (D9Mit54). Removing those yields an average F_ST_ of 0.14.

**Table 1:** **Geographic origin of samples and their genetic diversity parameters at 19 microsatellite loci**

**Region**	**Countries**	**Populations**	**sub-species**	**Latitude N**	**Longitude E**	**N**	**Hexp**	**Hobs**	**Average number of allele**	**References**
**Africa**	Kenya	Mombasa	castaneus	−3.93	39.75	8	0.72	0.52	6.58	[12]
		Nairobi	castaneus	−1.28	36.75	23	0.80	0.55	10.84	[12]*
	Madagascar	Malagasy	castaneus	−19.54	47.51	31	0.67	0.48	9.74	[15]*
	Morocco	Azemmour	domesticus	33.41	−8.03	19	0.74	0.66	7.37	[16]*
		Tanant	domesticus	31.80	−6.95	32	0.65	0.48	8.05	[16]
	Senegal	Dakar	domesticus	16.46	−15.69	10	0.68	0.52	5.11	[17]
	Spain	La Palma	domesticus	28.68	−17.85	30	0.75	0.67	9.68	[17]
	Tunisia	Kairouan	domesticus	35.67	10.10	12	0.82	0.60	10.53	[17]
		Tunisia Central-East	domesticus	35.66	10.73	40	0.83	0.50	13.68	[17]*
**Asia**	Armenia	Megri	musculus	39.90	46.24	10	0.75	0.67	7.16	[18]*
	Kazakhstan	Kazakhstan	musculus	43.00	77.00	46	0.74	0.63	13.32	[19]
	China	Ningxia	musculus	34.98	105.93	22	0.75	0.66	11.37	this study
		Xinjiang	musculus	43.47	84.89	6	0.69	0.64	5.47	this study
	Georgia	Abkhazia	musculus	43.12	41.27	5	0.69	0.68	5.11	[18]
		Adjaria	domesticus	41.58	41.66	3	0.64	0.75	3.95	[18]*
		East Georgia	musculus	41.70	45.22	32	0.80	0.70	15.26	[18]*
	India	India North	castaneus	28.10	77.27	30	0.84	0.63	15.37	[20]
		Nilgiri	castaneus	11.57	76.64	7	0.66	0.46	5.42	[20]*
	Iran	Ahvaz	domesticus	31.53	48.53	45	0.89	0.83	19.79	[21]
		Bandar-Abbas	“Central Iran”	27.86	56.30	34	0.81	0.61	13.21	[21]
		Birdjand-Zabol	“South East Iran”	31.54	61.14	14	0.81	0.61	10.37	[21]*
		Chabahar	“South East Iran”	25.60	60.79	53	0.77	0.55	12.26	[21]
		Hamedan	domesticus	35.05	48.88	29	0.76	0.71	9.37	[21]
		Iranshahr	“South East Iran”	27.36	60.25	29	0.77	0.48	9.42	[21]
		Iran North East	musculus	36.88	59.01	10	0.75	0.64	8.68	[12]
		Khakh-Qaene	musculus	33.95	58.84	21	0.81	0.64	13.47	[12]*
		Isfahan	“Central Iran”	32.77	51.58	30	0.76	0.68	9.95	[21]
		Tehran	“Central Iran”	35.70	51.42	5	0.67	0.74	4.53	[12]*
		Yazd	“Central Iran”	31.74	54.20	21	0.66	0.51	6.74	[12]
	Pakistan	Pakistan	castaneus	33.46	72.95	22	0.86	0.71	15.11	[20]*
	Thailand	Thailand	castaneus	13.95	100.57	12	0.82	0.54	10.42	[22]
	Turkmenistan	Turkmenistan	musculus	35.89	61.65	3	0.63	0.69	3.89	this study
**Europe**	Bulgaria	Bulgaria	domesticus	42.74	27.57	14	0.79	0.65	9.16	[21]
	Cyprus	Cyprus	domesticus	34.79	32.81	28	0.77	0.59	9.74	[23]*
	Czech Republic	Czech Republic	musculus	49.16	16.20	43	0.72	0.52	10.00	[24,25]
	France	Massif-Central	domesticus	44.38	3.00	44	0.76	0.60	11.42	[19]
	Germany	Koeln-Bonn	domesticus	50.88	6.88	36	0.79	0.54	11.21	[19]
	Italy	North Italy	domesticus	45.38	9.38	4	0.69	0.66	4.68	[21]*
	Russia	Moscow	musculus	55.76	37.62	5	0.51	0.58	3.26	[18]
	Spain	Spain	domesticus	42.08	−1.65	13	0.72	0.49	7.05	[17]
**Middle East**	Israel	Israel	domesticus	32.97	35.71	11	0.79	0.75	7.95	[17]*
	Lebanon	Amchit	domesticus	35.73	34.15	13	0.81	0.76	8.95	[17]
		Jbeil	domesticus	34.13	35.72	14	0.79	0.70	8.05	[17]
		Rayak	domesticus	33.87	36.03	23	0.79	0.72	8.95	[17]
		Terbol	domesticus	33.82	35.98	14	0.67	0.58	5.74	[17]
	Syria	Latakia	domesticus	35.52	35.79	4	0.61	0.58	3.42	[17]
	Turkey	Turkey East	domesticus	38.63	42.90	3	0.66	0.80	3.68	this study

N = number of individuals analysed, Hexp = expected heterozygosity, Hobs = observed heterozygosity, *samples kept as wild-derived inbred strains at the CGSS repository of Montpellier http://www.isem.univ-montp2.fr/recherche/les-plate-formes/conservatoire-genetique-de-souris-sauvages/presentation/.

### Correspondence analysis

**Figure 2:**
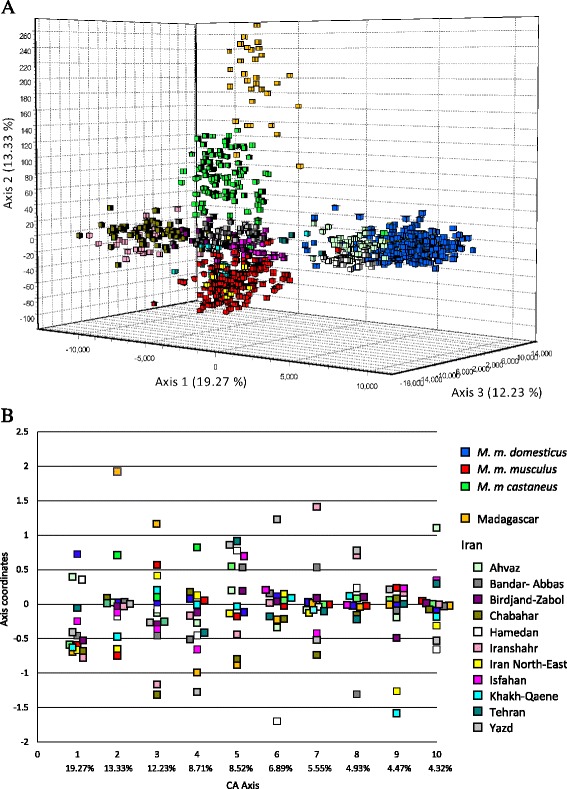
**Correspondence analysis. A)** 3D analyses on populations’ centroids, every square representing an individual. **B)** Axis coordinates for the first 10 axis of the analysis, every square representing a population centroid.

The Correspondence Analysis (CA) depicts the relative positions of individual genotypes projected onto the 3D space of maximal differentiation of each sample’s centroid (Figure 2A). We chose to represent the samples assigned to the three peripheral sub-species (*M. m. domesticus*, *M. m. musculus* and *M. m castaneus*) in previous studies by blue, red and green squares respectively, the Malagasy sample in orange and the Iranian samples with a palette of colours. The first three axes explained more than 44.8% of the total inertia. The coordinates of the centroids on the 10 first axes can be seen in Figure 2B. Contrary to what could have been expected *a priori*, we do not simply observe *M. m. domesticus*, *M. m. musculus* and *M. m. castaneus* separated by the two first axes and the central samples clustering somewhere in the middle, but rather there are two South East Iranian samples (Iranshahr and Chabahar) which pull the 1^st^ axis in a direction opposite to *M. m. domesticus*, while the differentiation between *M. m. musculus* and *M. m. castaneus* is only seen on axis 2. Interestingly, Malagasy animals which have been previously shown to possess a *M. m. gentilulus* mtDNA haplotype [15] are pulled further away along the *M. m. castaneus* cloud on axis 2, this possibly reflecting a founder effect in line with their somewhat lower diversity as reported above. Axis 3 primarily accounts for a clear opposition between South East Iranian and Malagasy samples. As expected Ahvaz and Hamedan, which have been shown to harbour predominantly *M. m. domesticus* matrilines [21], clustered with *M. m. domesticus* on one side and Khakh-Qaene and Iran North-East (a grouping of samples from the Mashhad region [26]) with *M. m. musculus*. The last samples predominantly from central Iran (Bandar-Abbas, Birdjand-Zabol, Isfahan, Tehran and Yazd) are situated at various distances in the middle of the axis 1, 2 and 3 (Figure 2A and B), but that of Yazd is strongly associated with the negative coordinates of axis 4 (Figure 2B). Other peculiarities can be found on the other axes and, even if no strictly private alleles were found for any particular sample except for very rare ones, these associations of different samples with different axes is indicative of the existence of groups of particular alleles among the 964 present in the global data set that pull the signal along theses axes (not shown).

### Population tree

**Figure 3:**
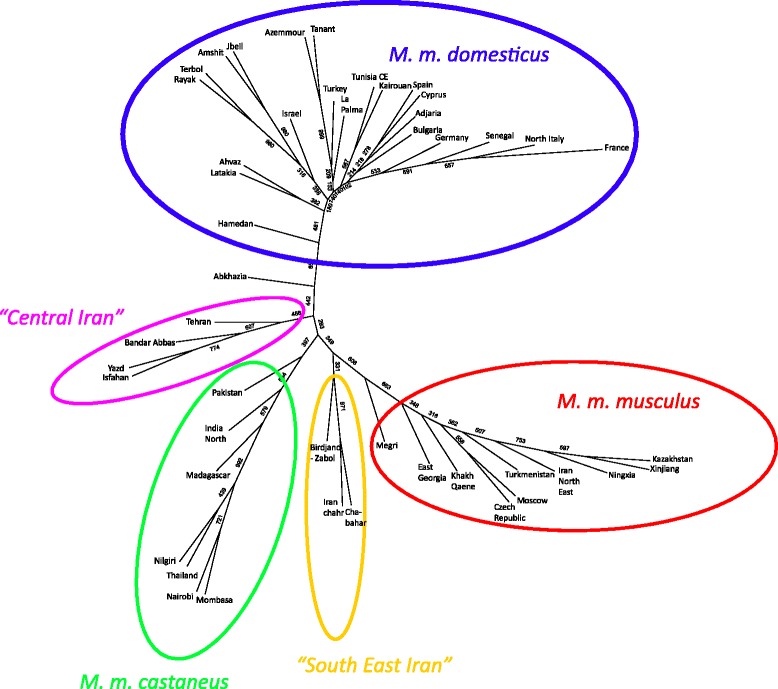
**Neighbour-joining tree based on Reynold’s distance, bootstrap values are indicated on every branch.**

A complementary graphical representation of population differentiation is provided by the Neighbour-Joining tree of Figure 3. The bootstrap values are not very high, but there is a high coherence in the phylogeographical groupings on the tree. The three major subspecies are clearly grouped together, with the only exception for the sample of Abkhazia (western Georgia) which did not cluster with *M. m. musculus,* while it was previously described from electrophoretical data as being predominantly of *M. m. musculus* composition (55% *M. m. musculus*, 45% *M. m. domesticus* –[18]). The Iranian mice other than those previously referred to *M. m. domesticus* or to *M. m. musculus* form two clearly distinct groups that are not closer to each other than they are from *M. m. castaneus*. One group called Central Iran (in pink) contains the samples from Tehran, Bandar-Abbas, Yazd and Isfahan and another group called South-East Iran (in yellow) contains samples from Birdjand-Zabol, Iranshahr and Chabahar. This last group was located opposite of *M. m. domesticus* on axis 1 of the CA and clearly stands on its own on the NJ tree.

### Individual allele-sharing distance tree

**Figure 4:**
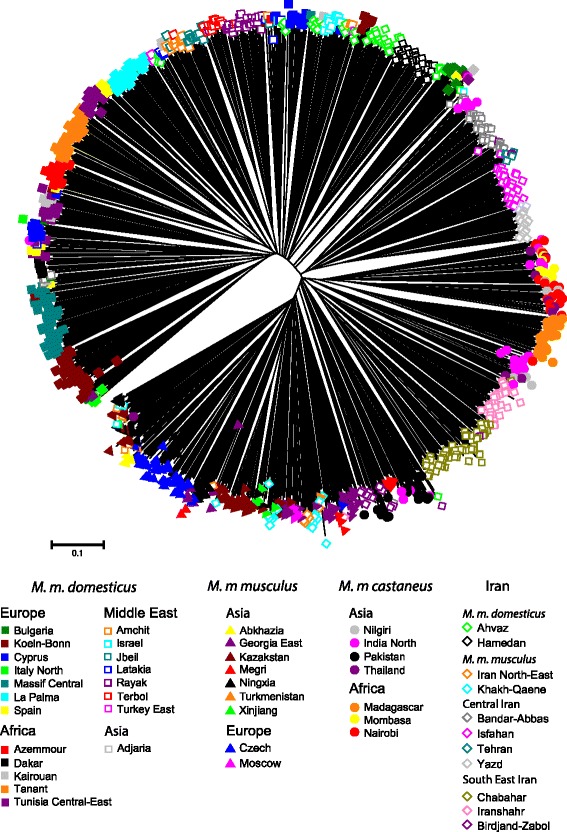
**Neighbour-joining tree based on the calculation of the proportion of shared alleles calculated for all individuals.** Samples from the same location share the symbol/colour pattern.

Representing nearly a thousand individuals on a single tree is difficult; nevertheless, the clustering obtained at the population level can largely be seen also at the individual level (Figure 4). Although the relative proximity of the different branches of the tree cannot be evaluated properly, it is remarkable that the order in which the various samples are organised fits almost perfectly with the population tree of Figure 3, starting with the *M. m. domesticus* samples at one point and ending with the ones referable to *M. m. musculus*. Remarkable also is the fact that most samples appear rather homogeneous. For instance, the individuals from South-East Iran: Iranshahr (light pink empty diamonds) and Chabahar (dark green empty diamonds) group together and are very close to each other. The same is seen in another sector of the tree with Yazd (light grey empty diamonds) and Isfahan (dark pink empty diamonds) and Bandar-Abbas (dark grey empty diamonds) from Central Iran. Interestingly, the Birdjand-Zabol samples (dark purple empty diamonds) superimposes with Pakistani (black filled circle) and some India North (pink filled circle) individuals, whereas it groups loosely with the South-East Iranian samples in the population tree and lies not far from the central Iranian ones in the CA. This was the sample showing the least coherence between the three types of analyses.

### Structure analysis

**Figure 5:**
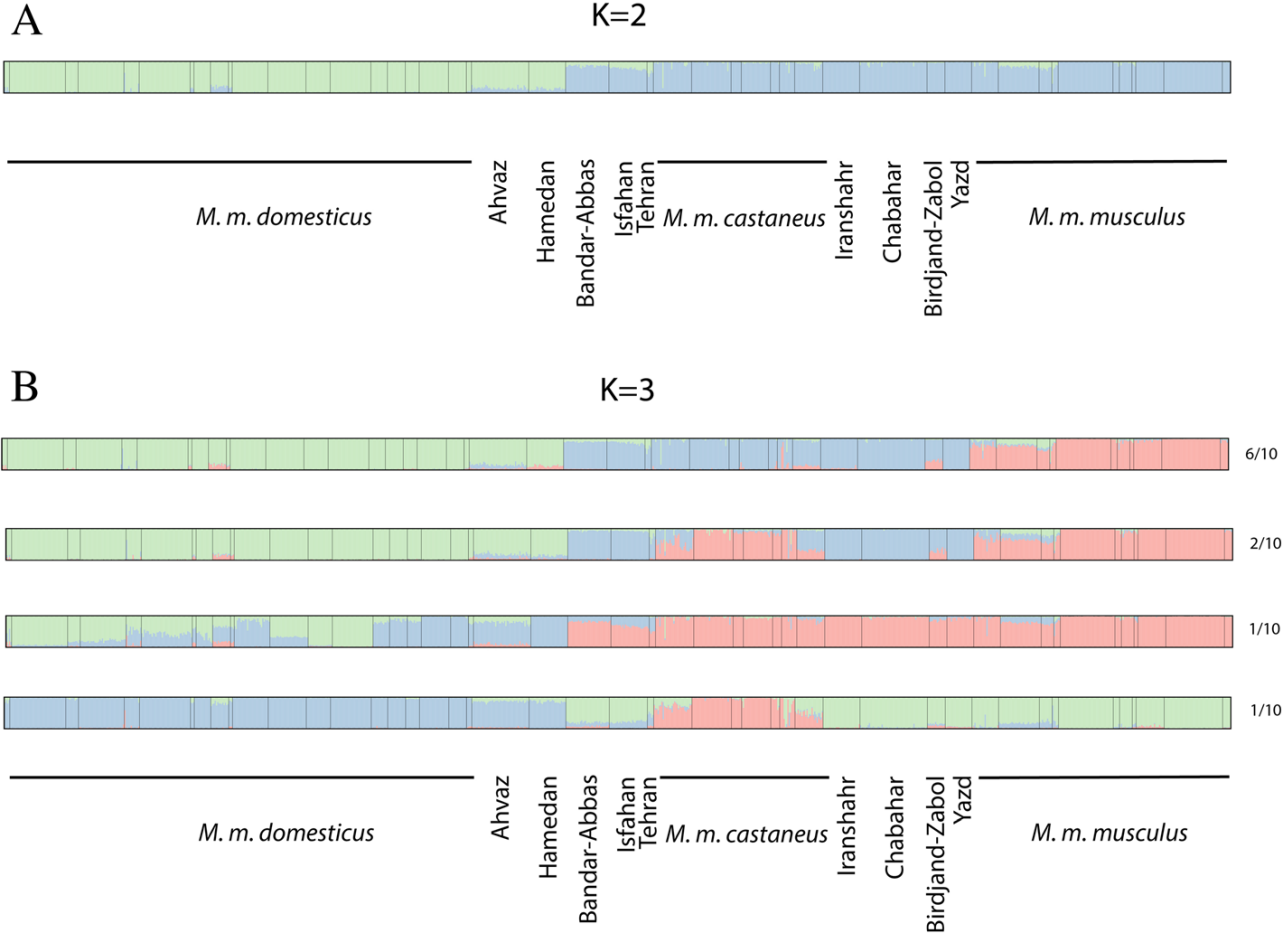
**Structure results for K = 2 (A) and K = 3 (B) of the mouse populations used in this study.** The different configurations found for K = 3 are represented as well as the number of times that the same pattern was obtained in 10 independent runs.

The Bayesian clustering procedure implemented in STRUCTURE [27] was performed with various numbers of partitions. Interestingly, the software was not able to converge for K larger than 2, and the standard deviation of the LogLikelihood among runs was quite high for larger values of K (Table 2). Accordingly, the criterion of Evanno et al. [28] (a steep change in the likelihood) or other more recently derived methods (see [29]) could not really be applied, the probable reasons for this are discussed further below. For this value of the partition, the samples were divided between *M. m. domesticus* on one side and non-*M. m. domesticus* on the other side, thus grouping *M. m. musculus*, *M. m. castaneus* and the Iranian samples (Figure 5A). All the runs for larger values of K yielded partially incompatible outputs, primarily because the assignment of Iranian samples varied from one run to the other. For K = 3, four different types of configurations were obtained (Figure 5B). The two groups of Iranian samples grouped either with *M. m. castaneus* (6 runs out of 10) or with *M. m. musculus* (1 run out of 10). Interestingly, in 2 runs out of 10, *M. m. musculus* and *M. m. castaneus* clustered together, leaving Central and South-East Iran forming one population. In the last configuration, all samples from Iran (except *M. m. domesticus*), *M. m. musculus* and *M. m. castaneus* clustered together while *M. m. domesticus* was divided in two groups: Eastern versus Western Mediterranean. For K = 4, six types of configurations were obtained, four of which separate the Iranian samples from the rest (see Additional file 1: Figure S1), two grouped Central and South East Iran with *M. m. castaneus*. Interestingly, these very samples were never grouped with *M. m. musculus* for K = 4, and some of the heterogeneity was carried by *M. m. domesticus* in three runs where possibly the occidental samples of this subspecies differentiated from the oriental ones. For K = 5 (see Additional file 1: Figure S2), a mixture of all these situations was obtained in several combinations with five different types of configuration. It is noteworthy, however, that the non-stable alternatives proposed by the software always turn around the same handful of clustering hypotheses. Further, for every of these values of K, some samples appeared to display detectable levels of introgression (see for instance Ahvaz, Thailand or Pakistan samples-Additional file 1: Figure S1).

**Table 2:** **Summary of STRUCTURE results as displayed with STRUCTURE Harvester**

**K**	**Reps**	**Mean LnP(K)**	**Stdev LnP(K)**	**Ln’(K)**	**|Ln”(K)|**	**Delta K**
1	10	−105767.55	0.47	NA	NA	NA
2	10	−97891.36	20.79	7876.19	4855.47	233.52
3	10	−94870.64	438.61	3020.72	775.75	1.77
4	10	−92625.67	312.34	2244.97	309.48	0.99
5	10	−90690.18	336.53	1935.49	NA	NA

## Discussion

### Overall differentiation

As was to be expected, the population samples from the best described subspecies differentiate clearly from each other, and can be unambiguously assigned to *M. m. musculus*, *M. m. domesticus* or *M. m. castaneus* in keeping with previous studies (see [5] for a review). These long recognised entities are separated by the three first canonical axes of the CA in Figure 2, and constitute the three longest branches of the distance tree in Figure 3 and occupy different sectors of the circle in the allele-sharing tree of Figure 4. For the sake of comparison with the literature, an AMOVA performed among these three entities only indicated a F_CT_ value of 0.70 for an average within group differentiation of F_SC_ = 0.12.

When calculated inside each of our sampling of the peripheral subspecies, this becomes 0.10, 0.13 and 0.09 for *M. m. castaneus*, *M. m. domesticus* and *M. m. musculus* respectively, thus indicating a consequent amount of internal variation inside each of them. However, when the whole collection was submitted to the Bayesian assignation of STRUCTURE, only the partition between *M. m. domesticus* and all the rest appeared stable (Figure 5A).

One noticeable point is that there is a quite good geographical coherence in each group. If one considers *M. m. domesticus* for instance, the fan-shaped sub-tree of Figure 3 separates well the Near-East samples from the European ones. The Senegalese sample appears clearly as a recent offshoot of European origin, while this is not the case for Northern-African ones (Morocco and Tunisia). For *M. m. musculus*, the feather-like sub-tree indicate remote branching for the Chinese and Kazak samples, a fact to be put in relation with the probable recent eastwards expansion of this subspecies from the Caucasus region [26,30]. As to the *M. m. castaneus* cluster, it is the one where unexpected assignations occur. As reported above, the Malagasy sample clearly belongs to this group despite it possessing *M. m. gentilulus* mtDNA. This is another example of the possibility for maternal lineage capture during an expansion process. Madagascar is thought to have been populated by mice quite recently (ca 1,000 years, [15]) with the development of trade between India and Indonesia (were the nuclear genome most likely comes from), the Arabic peninsula (for the *M. m. gentilulus* maternal lineage [11]) and Africa. Of interest also is the position of the Kenyan mice, both from the coast (Mombasa) and the interior (Nairobi) which appear also as a recent *M. m. castaneus* offshoot, despite harbouring a mixture of *M. m. castaneus* and *M. m. domesticus* matrilines [17] as well as supplementary matrilines (see Additional file 2). Another noticeable point is, as expected, that samples known to be introgressed as the above-mentioned Abkhazian one, tend to “move” toward the centre of the tree. This is also the case for the Armenian sample from Megri, which is *M. m. musculus* for 2/3 of its nuclear genome [18] and was already shown to be under multiple genetic influences since it also contains for instance at the ABP locus three different alleles, each one ascribed to a given subspecies [31].

### Status of Iranian plateau populations

The most interesting results of this study concern the samples of the Iranian plateau, which have sometimes been coined “central populations” because of many uncertainties as to their taxonomic affiliation. Putting aside the samples unambiguously referable to *M. m. musculus* (Khakh-Qaene and Iran North-East) and *M. m. domesticus* (Ahvaz and Hamedan), we are left with two clearly separated entities as described in the Results section. Interestingly, this fits rather well with the mitochondrial description recently provided [12] where each of the entities recognised in Iran is associated predominantly with one haplogroup (HG) essentially not found elsewhere. Namely, when recompiling the aforementioned data [12] for the populations’ samples of this study, the Central Iran group is associated with mitochondrial haplogroup Hg1B (78%) and South-East Iranian is associated with haplogroup Hg3 at 88%, while these two HGs are practically non-existent outside Iran where *M. m. castaneus* is almost completely associated with Hg2. The mitochondrial tree of all studied populations is provided in Additional file 1: Figure S3 to illustrate this point. As discussed by Rajabi-Maham et al. [12], the separate coalescence of these phylogenetically independent haplogroups have most likely arisen in allopatry during past periods of geographical isolation, and our nuclear genome results fit quite well with this view as they also point towards the existence of two independent groups in Iran, one in the center, and one in the South-East. These two groups are loosely related to *M. m. castaneus*, as are their matrilines (Additional file 1: Figure S3). Furthermore, the separation of the different entities in Iran seems to fit the topography of the country. *M. m. domesticus* samples are separated from the Central Iran individuals by the Zagros Mountains, Central Iran from *M. m. musculus* through the Kavir desert and from south-east Iran through the Lut desert (Figure 1B).

The central and south-east Iranian phylogroups are as distinct from each other as they are from the south-east Asian *M. m. castaneus*, even if many signs of admixture and secondary contacts are to be found, be it on the nuclear genome or the mitochondrial data. This is likely the principal reason why the STRUCTURE clustering did not converge to stable partitions for values of K larger than 2; probably because certain loci may have been more prone to be exchanged than other after secondary exchange. Hence, the present genomic make-up of Iranian populations is likely to be a mosaic of locally evolved haplotypes (like the mitochondrial matrilines) and segments imported from their neighbours in proportions that remain to be estimated. Nevertheless, they necessarily have evolved as geographical isolates during a certain amount of time and cannot be considered as resulting from simple admixture of surrounding populations. Hence, we may consider them as new independent entities, even if monophyly will rarely be achieved because of the retention of ancestral polymorphisms and secondary gene flow.

Attempts at setting the time frame for these exchanges with ABC methods have been recently performed [32], but these did not formally consider the existence of independent Iranian entities. The ancient origin of most Iranian phylogroups has however been recently reinforced by independent data showing they may have retained ancestral morphological features [33]. Only more sophisticated model-based analyses relying on genome-wide multilocus data will be able to tell what kind of genetic exchanges they are still able to maintain. Such genome-wide data exist currently solely for the comparison between the peripheral *M. m. domesticus* populations in Germany and France, versus *M. m. musculus* in Czech Republic and Kazakhstan [34] and between wild-derived lines representing each of the three peripheral subspecies [35]. These studies have revealed complex patterns of mutual introgression but cannot enlighten us as to the history and status of the Iranian phylogroups.

From the taxonomical standpoint, this situation deserves further scrutiny, since the distinctiveness of these three phylogroups should prevent the use of a single Latin trinomial like *M. m. castaneus*. However, solving the problem would require having access to hypothetical voucher specimens and a better delineation of present day limits of these phylogroups. This is not an easy task, because the species as a whole has undergone a phase of post-glacial expansion triggered by its association with Neolithic humans which may have induced a phase of complex hybridization, introgression and de-differentiation. This de-differentiation reaches some clear limits when distantly related subspecies like *M. m. domesticus* and *M. m. musculus* are concerned since their contact amounts to hybrid zones with restricted gene flow (see for instance [36] and the literature cited therein), but is not so clear what happens between the four non-*domesticus* entities considered here.

## Conclusion

Our analysis of a Eurasian collection of wild mice encompassing both peripheral populations and a large number of central ones is the first comprehensive approach aiming at providing a global view of the relationships among the taxa constitutive of this complex subspecific assemblage. We propose here that the Iranian plateau is home to two more units displaying complex primary and secondary relationships with their long recognized neighbours *M. m. domesticus*, *M. m. musculus* and *M. m. castaneus*. If one adds to this the *M. m. gentilulus* from the Arabic peninsula and the new clade described from Nepal [13], the taxonomic diversity is clearly higher than previously thought. This is not a surprise if one considers the highly tormented landscape of the central regions where the complex species originated from, where there was clearly space for more than three geographical isolates before its worldwide expansion. The fact that this happens in and around the Iranian plateau is consistent with the key geographical position of this region which constitute an obligate passage between the lowlands of central Asia to the North, the Fertile Crescent and the Near-East to the West, and the Indian subcontinent to the East. Although the present-day distribution of distinct phylogroups does not necessarily indicate where the ancestral species was originally located, it becomes clear from the above results that the Iranian plateau should be included in the candidate regions together with the Indo-Pak sub-continent and neighboring Afghanistan. Indeed, in this large Middle-Eastern region, no less than six distinct phylogroups have differentiated and presently interact with each other. This should now be taken into account when building evolutionary scenarios.

## Methods

### Mice sampling

Most of the individuals included in this study come from the DNA collection established at ISE-M between 1985 and 2005 with the exception of the samples from Massif Central, Cologne-Bonn, Czech Republic and Kazakhstan that come from the collection held at the MPI-Plön [19]. All these samples have been described and used in previous publications, the references of which are given in Table 1, together with their geographical locations.

### Genotyping

19 microsatellites were chosen from previous studies: PP8E11 (Chr2), PP6E09 (Chr3), D1EnsmusG22992 (Chr3),PP10E08 (Chr4), D5Mit149 (Chr5), D6Mit309 (Chr6), PP10A02 (Chr8), D9Mit54 (Chr9), D9Mit330 (Chr9), PP3A02 (Chr10), PP4A02 (Chr11), D13Mit61 (Chr13), D14Mit203 (Chr14), E1EnsmusG46849 (Chr15), D15Mit98 (Chr15), PP7B08 (Chr16), PP8A05 (Chr17), D19Mit39 (Chr19) and, PP2A01 (ChrX) [37,38]. Forward primers were labeled with FAM or HEX dye on the 5’end and the reaction conditions were as follow: denaturation, 95°C for 15 min, 28 cycles with 0.30s at 95°C, 1.30 min at 60°C and 1.30 min at 72°C, final elongation 10 min at 72°C. Primers (Additional file 1: Table S1) were pooled in 5 different reactions with a final reaction volume of 5 μl using Multiplex PCR kit (Qiagen). PCR products were then diluted 1/20 in Millipore water. 1 μL of this dilution was added to a mix of 0.1 μL ROX Standard (Applied Biosystems) and 10 μL HiDi Formamid. Heating for the denaturation step was 2 min at 90°C followed by 5 min at 20°C. Microsatellites were scored using GeneMapper (Applied Bioscience).

### Data analysis

Basic diversity parameters were obtained using Genetix [39] and Arlequin [40]. Several ways of dissecting the genetic variability present at the 19 microsatellites loci were used concurrently. We first performed a Correspondence Analysis (CA) using the AFC-3D procedure of Genetix. This is an unsupervised method which allows representation of the differentiation of samples along independent factorial axes. We did this by taking the centroids of samples as active elements (and individuals as supplementary elements) considering alternatively the whole collection (47 population samples, 963 individuals) with those samples previously described unambiguously as belonging to the peripheral subspecies, either *M. m. domesticus* (22 populations, 367 individuals), *M. m. musculus* (11 populations, 172 individuals) or *M. m. castaneus* (7 populations, 133 individuals) considered individually, or the samples from Iran (11 populations, 291 individuals) including individuals from all the different sub-species.

The pairwise Reynold’s distances were computed using the Gendist program of the Phylip package [41] and 1,000 bootstrapped datasets were generated with Seqboot, followed by the Neighbor and Consense procedures from the same package to obtain a Neighbour-Joining consensus population tree. Additionally, an Allele Sharing distance tree considering all individuals was calculated using the software Populations 1.2.32 [42] and visualized with MEGA 6 [43].

Finally, we used the STRUCTURE program [27] to assign the collection of individuals to several putatively reproductively independent groups. The run parameters were as follow: a burn-in period of 1,000,000 simulations followed by a run length of 1,000,000 MCMC simulations and ten iterations for K (number of clusters) equals 1 to 5. The runs were performed using the admixture model with the Loc Prior option. Results were summarized using Structure Harvester [44]. K was chosen using the criterion of Evanno et al. [28]. To draw the structure diagrams the softwares CLUMPP [45] and Distruct [46] were used.

The mitochondrial D-loop sequences tree from Additional file 1: Figure S3 was produced with MEGA6 [43]. The inference was performed according to the maximum likelihood method based on the Tamura-Nei model [47]. The boostrap values (150 replicates) are shown. Initial tree(s) for the heuristic search were obtained by applying the Neighbor-Joining method to a matrix of pairwise distances estimated using the Maximum Composite Likelihood (MCL) approach. A discrete Gamma distribution was used to model evolutionary rate differences among sites (5 categories (+G, parameter = 0.1440)). The rate variation model allowed for some sites to be evolutionarily invariable ([+I], 62.0668% sites). The tree is drawn to scale, with branch lengths measured in the number of substitutions per site. The analysis involved 867 nucleotide sequences. All positions with less than 95% site coverage were eliminated. That is, fewer than 5% alignment gaps, missing data, and ambiguous bases were allowed at any position. There were a total of 849 positions in the final dataset.

### Availability of supporting data

The microsatellites dataset supporting the results of this article on Dryad: doi:10.5061/dryad.ck276.

## Additional files

Additional file 1:
**Figure S1.** Structure results for K=4. The different configurations found are represented as well as the number of times that the same pattern was obtained in 10 independent runs. **Figure S2.** Structure results for K=5. The different configurations found are represented as well as the number of times that the same pattern was obtained in 10 independent runs. **Figure S3.** Mitochondrial D-loop sequences tree. An expanded version of it is available in Additional file 2. **Table S1:** Primers used to amplify the 19 microsatellites used in this study. Additional file 2: Enlarged rectangular tree of Additional file 1: Figure S3 above.
