# Supplementary material for: Eurasian house mouse (Mus musculus L.) differentiation at microsatellite loci identifies the Iranian plateau as a phylogeographic hotspot
Source: BMC Evol Biol. 2015 Feb 25;15:26. doi: 10.1186/s12862-015-0306-4 (PMC4342898; doi:10.1186/s12862-015-0306-4)
Supplement: Additional file 1: — Figure S1. Structure results for K=4. The different configurations found are represented as well as the number of times that the same pattern was obtained in 10 independent runs. Figure S2. Structure results for K=5. The different configurations found are represented as well as the number of times that the same pattern was obtained in 10 independent runs. Figure S3. Mitochondrial D-loop sequences tree. An expanded version of it is available in Additional file 2. Table S1: Primers used to amplify the 19 microsatellites used in this study. [file 12862_2015_306_MOESM1_ESM.docx]

**Additional file 1**

Figure S1

Structure results for K=4. The different configurations found are represented as well as the number of times that the same pattern was obtained in 10 independent runs.


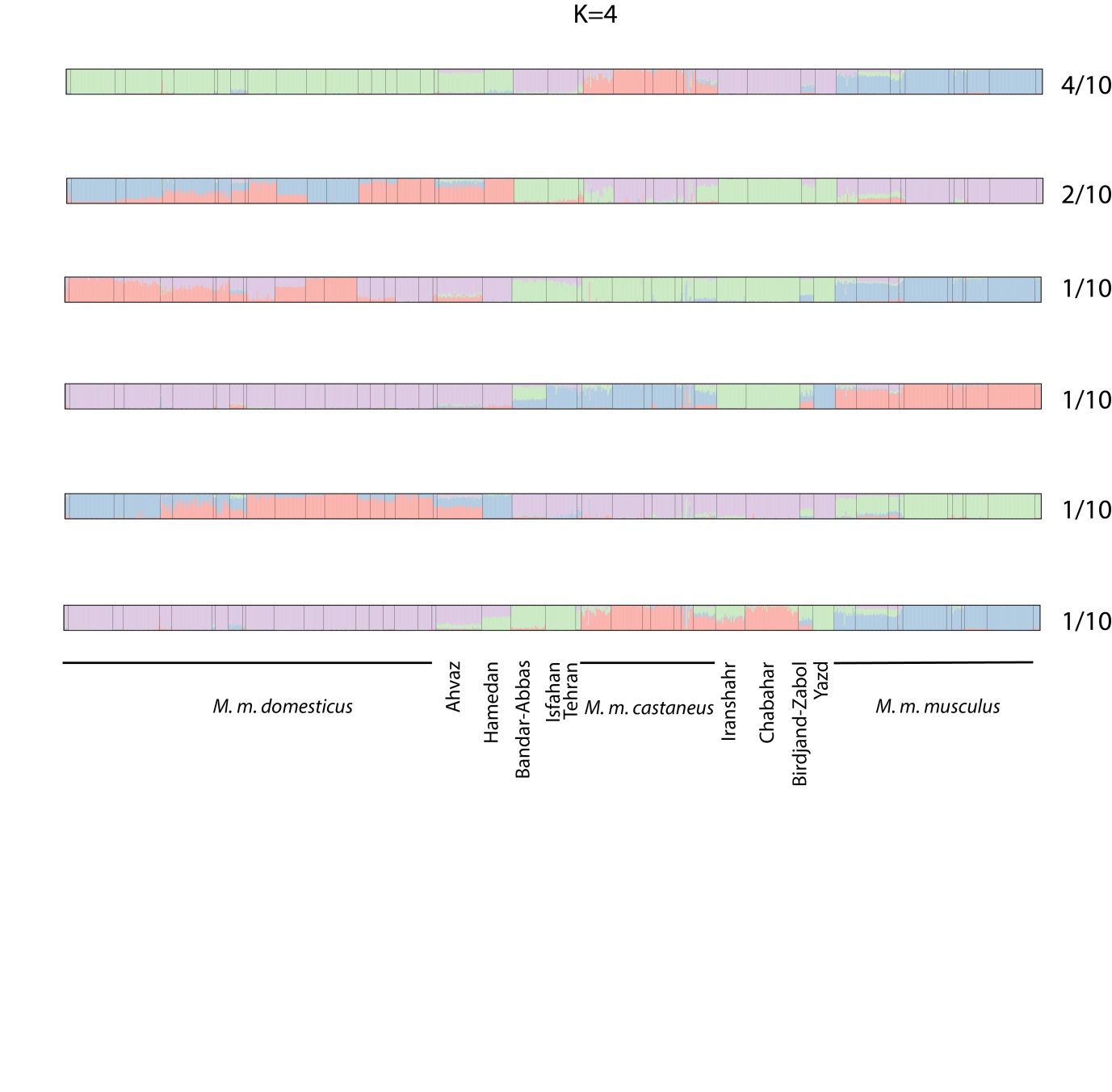


Figure S2

Structure results for K=5. The different configurations found are represented as well as the number of times that the same pattern was obtained in 10 independent runs.
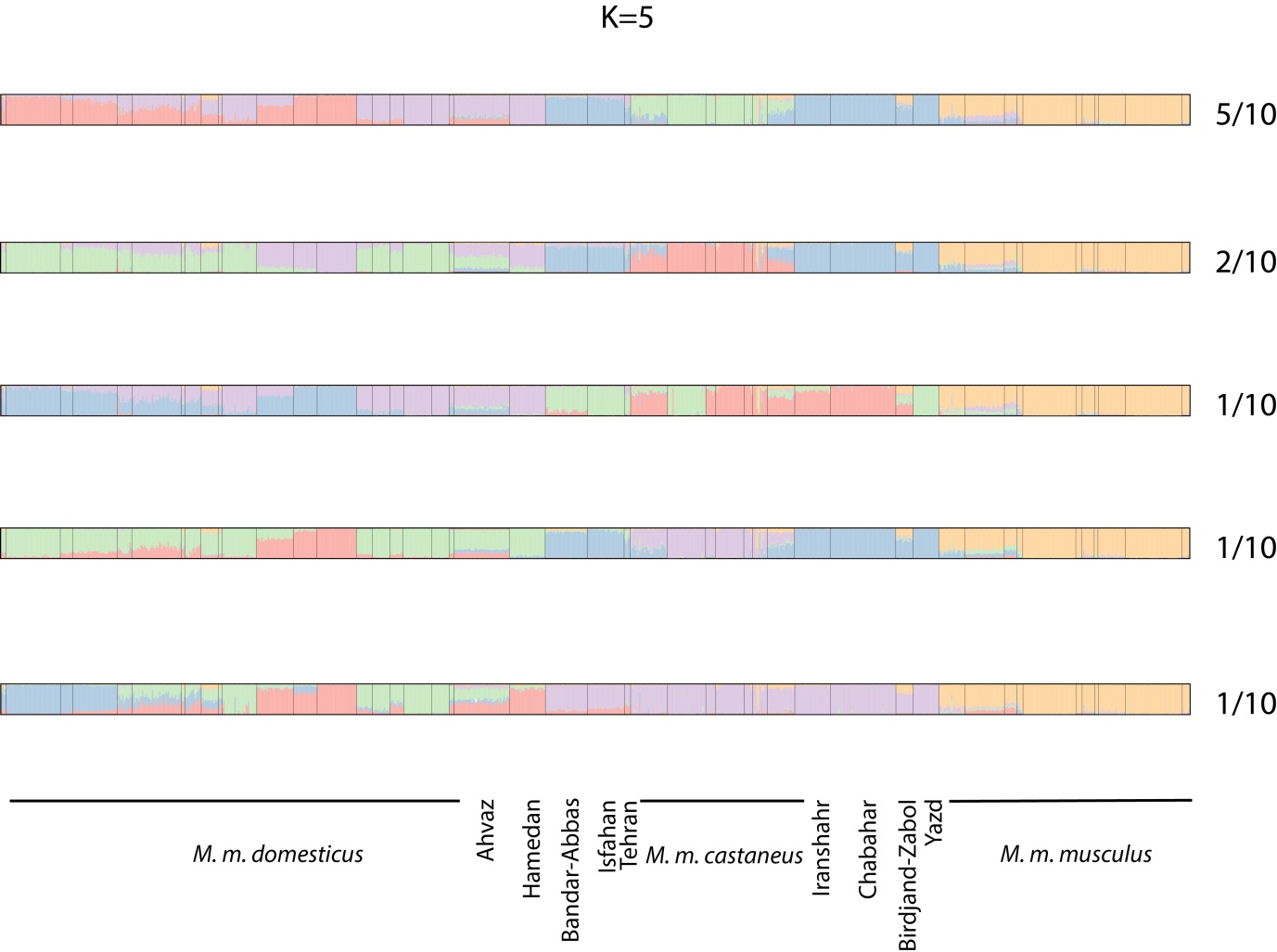


Figure S3:

Mitochondrial D-loop sequences tree produced with MEGA6 [46].The inference was performed according to the maximum likelihood method based on the Tamura-Nei model [47]. The boostrap values (150 replicates) are shown. Initial tree(s) for the heuristic search were obtained by applying the Neighbor-Joining method to a matrix of pairwise distances estimated using the Maximum Composite Likelihood (MCL) approach. A discrete Gamma distribution was used to model evolutionary rate differences among sites (5 categories (+G, parameter = 0.1440)). The rate variation model allowed for some sites to be evolutionarily invariable ([+I], 62.0668% sites). The tree is drawn to scale, with branch lengths measured in the number of substitutions per site. The analysis involved 867 nucleotide sequences. All positions with less than 95% site coverage were eliminated. That is, fewer than 5% alignment gaps, missing data, and ambiguous bases were allowed at any position. There were a total of 849 positions in the final dataset.

An expanded version of the same tree is given in the accompanying supplementary jpg file


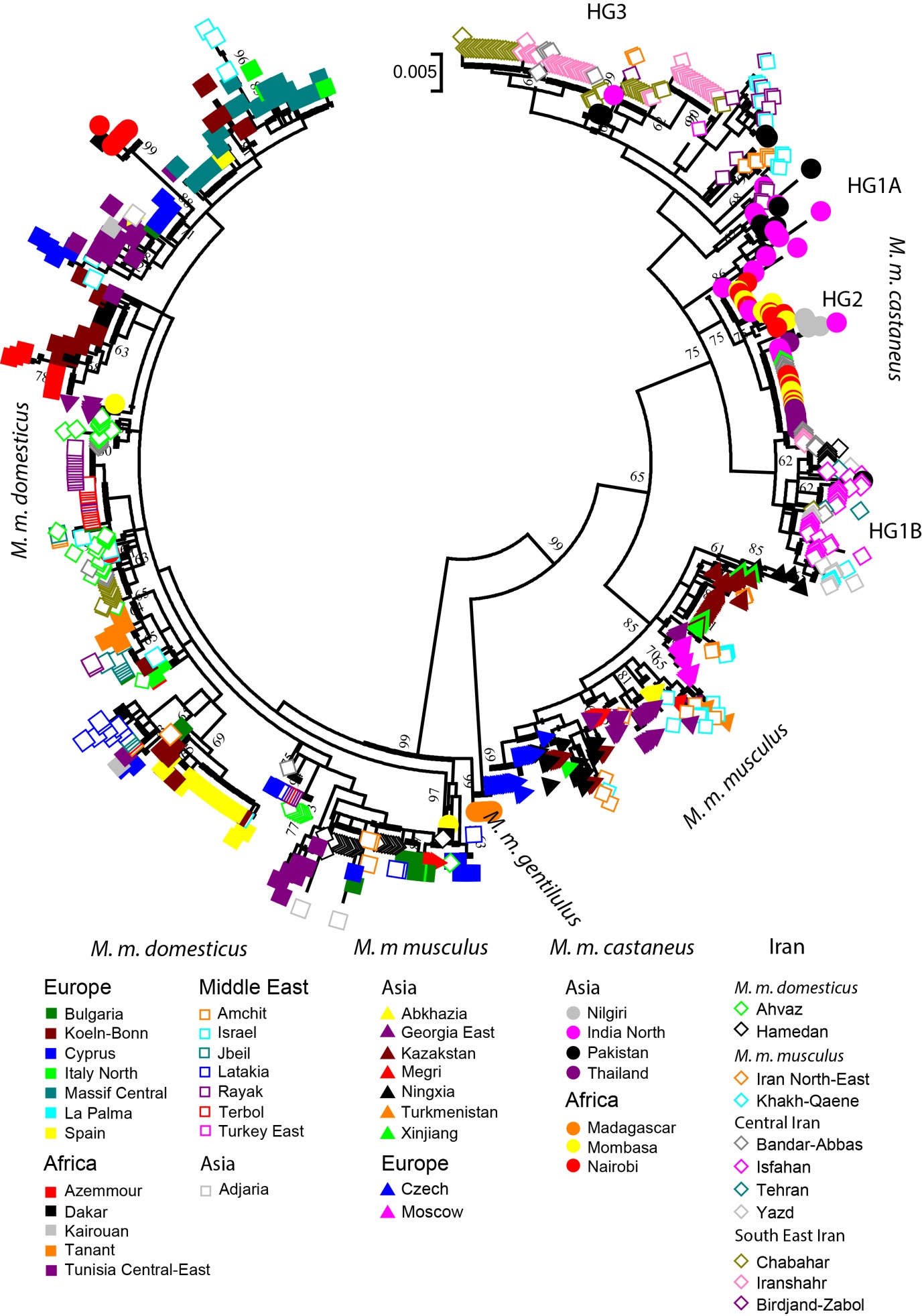


Table S1: Primers used to amplify the 19 microsatellites used in this study

| Forward primer | Sequence | Reverse primer | Sequence |
| --- | --- | --- | --- |
| PP8E11_F | actcgtgagacacacagtcctg | PP8E11_R | gtgtcagccaacaggcacttgc |
| PP10E08_F | aggctccaccacggagcacctc | PP10E08_R | ggaggttgagtccagcctagtc |
| PP10A02_F | agaaagcagcttacagtcccag | PP10A02_R | tggattgggatgagccttgaac |
| PP3A02_F | gccgatagccttgctctggctg | PP3A02_R | ccacttggtaaggtgtccatgc |
| PP4A02_F | tccagtcccagttgccagacac | PP4A02_R | aacctggtctcagaggctgtcg |
| PP7B08_F | actagcagagccacttcgggag | PP7B08_R | tcccaggatcgtccagctcagg |
| PP8A05_F | tctatcagatgggctctgggag | PP8A05_R | tcatccatgcaaagtaggcctc |
| PP6E09_F | gaggccaagtgtccagcagtgg | PP6E09_R | accaatctgcatgccatcatgg |
| D13Mit61_F | TGCTCCAATACAACAAGGTCC | D13Mit61_R | CCAGCCAAGGTGTGTTGAC |
| D14Mit203_F | GTTAGCCAATTTAGAGGAGAGCC | D14Mit203_R | CAGAACTCCAGTCTAACTATCACACA |
| D15Mit98_F | AGCACATTCTCCCAACAACC | D15Mit98_R | CAAAACAAGCACAAAACAAATACA |
| D19Mit39_E | GGAGGTCTCAGGAAATATTACTCC | D19Mit39_R | ATTCCTGTGTAAAGGTGGATGG |
| D5Mit149_F | TCAGGAAGTGATCTTCCAACG | D5Mit149_R | ATCTGATGCCTCTGACCTTCA |
| D6Mit309_F | TATGCTTTTTTTCAAATCTGTTGC | D6Mit309_R | CACTAGGAAACCCACCCTGA |
| D9Mit330_F | GAAATGAGGCTACTTACCACGG | D9Mit330_R | GATATGTACATTCAGATGCATCCA |
| D9Mit54_F | TGGGGATACTATGCCTTCTACTG | D9Mit54_R | CAGGTCAAGGCTACTTTTATTTTC |
| PP2A01_F | actctcaggaatgtgttgcgtc | PP2A01_R | tggagagaccttgggctaccca |
|  |  |  |  |
|  |  |  |  |
|  |  |  |  |
